# Impact of Dietary Trajectories on Obesity and Dental Caries in Preschool Children: Findings from the Healthy Smiles Healthy Kids Study

**DOI:** 10.3390/nu13072240

**Published:** 2021-06-29

**Authors:** Narendar Manohar, Andrew Hayen, Jane A. Scott, Loc G. Do, Sameer Bhole, Amit Arora

**Affiliations:** 1 School of Health Sciences, Western Sydney University, Penrith, NSW 2751, Australia; drnarendar@gmail.com; 2 Health Equity Laboratory, Campbelltown, NSW 2560, Australia; 3 Australian College of Physical Education, Sydney Olympic Park, NSW 2127, Australia; 4 School of Public Health, Faculty of Health, University of Technology Sydney, Ultimo, NSW 2007, Australia; andrew.hayen@uts.edu.au; 5 Discipline of Nutrition and Dietetics, School of Population Health, Curtin University, Perth, WA 6102, Australia; jane.scott@curtin.edu.au; 6 School of Dentistry, Faculty of Health and Behavioural Sciences, University of Queensland, Brisbane, QLD 4072, Australia; l.do@uq.edu.au; 7 Australian Research Centre for Population Oral Health, University of Adelaide, Adelaide, SA 5005, Australia; 8 Oral Health Services, Sydney Local Health District and Sydney Dental Hospital, Surry Hills, NSW 2010, Australia; sameer.bhole@health.nsw.gov.au; 9 Sydney Dental School, Faculty of Medicine and Health, The University of Sydney, Surry Hills, NSW 2010, Australia; 10 Translational Health Research Institute, Western Sydney University, Campbelltown, NSW 2560, Australia; 11 Discipline of Child and Adolescent Health, Faculty of Medicine and Health, Sydney Medical School, The University of Sydney, Westmead, NSW 2145, Australia

**Keywords:** diet, dietary trajectories, dietary patterns, overweight, obesity, early childhood caries, ECC, dental caries, health risk, preschool children

## Abstract

This study examines the impact of longitudinal dietary trajectories on obesity and early childhood caries (ECC) in preschool children in Australia. Mother–infant dyads from the Healthy Smiles Healthy Kids study were interviewed at 4 and 8 months, and 1, 2, and 3 years of age. Children underwent anthropometric and oral health assessments between 3 and 4 years of age. Multivariable logistic regression and negative binomial regression analysis were performed for the prevalence of overweight and obesity, and the number of tooth surfaces with dental caries, respectively. The intake of core, discretionary, and sugary foods showed distinct quadratic (*n* = 3) trajectories with age. The prevalence of overweight or obesity was 10% (*n* = 72) and that of early childhood caries (ECC) was 33% (mean decayed, missing, and filled tooth surfaces (dmfs) score: 1.96). Children with the highest trajectories of discretionary foods intake were more likely to be overweight or obese (adjusted OR: 2.51, 95 %CI: 1.16–5.42). Continued breastfeeding beyond 12 months was associated with higher dmfs scores (adjusted IRR: 2.17, 95 %CI: 1.27–3.73). Highest socioeconomic disadvantage was the most significant determinant for overweight or obesity (adjusted OR: 2.86, 95 %CI: 1.11–7.34) and ECC (adjusted IRR: 2.71, 95 %CI: 1.48–4.97). Targeted health promotion interventions should be designed to prevent the incidence of two highly prevalent conditions in preschool children.

## 1. Introduction

Obesity and early childhood caries (ECC) are the two common and important health problems affecting Australian children [1,2]. In Australia, 20% of children aged 2–4 years are either overweight or obese [3] and 34% have ECC by the age of 5-years [4]. Both these conditions can have detrimental long-term health consequences [5,6] and also have a tendency of progression into adulthood [1,5]. 

Childhood obesity and ECC are multi-factorial in origin with a diverse range of risk factors [7]. Recent evidence shows that these two conditions share common risk factors including low socioeconomic status (SES), poor diet, and other social–environmental factors [8,9]. Hence, considering the evident association between these two conditions, the Common Risk Factor Approach (CRFA) seems to be the most suitable interventional strategy as it seeks to target risk factors that are common to both the conditions [10].

Poor diet, comprising of energy-dense, low-nutritious foods, and an earlier introduction of such foods can contribute to the rising prevalence of childhood obesity and ECC [11,12]. For example, earlier research from the Healthy Smiles Healthy Kids (HSHK) study [13,14,15] identified that 95% of infants were introduced to discretionary foods, and more specifically, almost 43% of infants were introduced to sugar beverages (SSBs) before the recommended age of 52 weeks [16]. The research team further noted that over 13% and over 76% of Sydney infants were introduced to solid foods before 17- and 26-weeks post-partum, respectively [15]. Another Australian cohort study found that a high consumption of sugary drinks led to obesity and ECC in young children [9]. In addition to sugars, poor diet comprises of other constituents that are considered to be obesogenic (e.g., ultra-processed foods high in saturated and trans fats and/or salts) and cariogenic (e.g., acidic foods or beverages) [17,18]. Contrariwise, an individual’s diet also consists of foods that can have an obesity-protective effect (e.g., fruits and vegetables) [19]. Therefore, in order to understand the disease epidemiology and prevention of obesity and dental caries in early life, it is important to examine the impact of whole diet, rather than single nutrients or foods. Measuring multiple dietary elements together and their patterns assists in identifying the synergistic and correlational nature of individual foods and nutrients. Moreover, they may be suggestive of how individuals eat and in what frequency [20]. Furthermore, measuring diet patterns longitudinally (from early infancy to preschool age) will help with defining which foods and/or nutrients are amenable to change and at what stage of life [21], particularly if those foods or nutrients are specifically related to disease outcomes.

Dietary patterns in children have been examined using statistical approaches such as factor analysis, principal component analysis (PCA), and cluster analysis [22,23,24]. In recent years, the Group-Based Trajectory Modelling (GBTM) approach has emerged for examining longitudinal dietary patterns [25]. The GBTM identifies clusters of individuals who follow similar trajectories over time [26]. This study is innovative and novel, since it examines the dietary intakes evolving over the first three years of life using advanced statistical approaches such as GBTM and their impact on two subsequent health outcomes. This may provide a greater understanding of the diet–disease relationship, which in the case of the present study is obesity and ECC. 

Dietary patterns and their association with later health outcomes in children have been investigated previously [27,28]. However, studies exploring the association between dietary patterns and obesity amongst children [28,29] as well as the relationship between dietary patterns and ECC [28,30] have shown inconsistent findings. A recent Australian cohort study did not find any meaningful association between two dietary patterns (i.e., healthy and unhealthy), body mass index (BMI) scores, and ECC in toddlers [28]. Whilst a Singapore-based study [31] involving a multi-ethnic Asian children population, using multi-level mixed modelling to examine the dietary trajectories between 6 and 12 months of age and ECC at a later age (i.e., age 2 and 3 years), demonstrated an inverse relationship between healthy diet patterns and ECC [31]. It is suggested that inconsistencies in the limited existing evidence may be due to discrepancies in the methodological approaches used to examine dietary patterns [28]. Moreover, to the best of our knowledge, no study has used GBTM to examine the longitudinal dietary patterns of core, discretionary, and added-sugar foods in infancy and early childhood and their association with later health outcomes. Therefore, the aim of this study was to examine the association between healthy and unhealthy dietary trajectories and obesity and ECC in preschool children. Moreover, the use of GBTM to characterise dietary patterns in early life would assist in providing a more detailed picture of diet and its evolution with age. Hence, the objectives of this study were:

1. To investigate the impact of longitudinal dietary trajectories on obesity and ECC in Australian preschool children.
2. To ascertain the impact of sociodemographic, socioeconomic, behavioural, and biological factors on obesity and ECC in Australian preschool children.

## 2. Methods

### 2.1. Data Source

This study is a secondary analysis of data collected from an ongoing birth cohort study called Healthy Smiles Healthy Kids (HSHK) [32], which has followed socioeconomically diverse families based in South West Sydney (SWS) since 2009, as described in detail in earlier studies [15,32,33]. In terms of recruitment, Child and Family Health Nurses (CFHNs) recruited mother–infant dyads (*n* = 1035) between October 2009 and February 2010 from public hospitals located within the Sydney and South Western Sydney Local Health Districts (LHDs) (formerly known as Sydney South West Area Health Service). The mothers were provided information on the study at the first post-natal visit, and written consent was obtained. Interpreter services and written material in the native language of non-English speaking participants (i.e., Arabic, Assyrian, Hindi, Cambodian, Cantonese, Mandarin, Vietnamese, and Samoan) were also arranged to facilitate participation. 

### 2.2. Data Collection

#### 2.2.1. Dietary Data

Children’s dietary data were periodically collected via telephone interviews at five age points i.e., 4 months, 8 months, 1 year, 2 years, and 3 years, respectively. The dietary questionnaire was adapted from the Iowa Fluoride study [34], the NSW Child Health Questionnaire [35], the National Child Oral Health Survey [36], the Perth Infant Feeding Studies (PIFS I and II) [37,38], and the HSHK pilot study [39]. Children’s dietary habits, in terms of consumption of 32 individual food and drink items in the preceding seven days, were recorded using a short food frequency questionnaire (FFQ) (Table S1). At every interview, mothers were asked an open-ended question “In the past 7 days, how often was your baby/child fed each of the following foods and/or drinks?”. A numerical response was recorded to represent the number of times the specified food and/or drink was consumed in a week.

For dietary trajectory analyses, the 32 listed food and drink items were broadly categorised into ‘core’ and ‘discretionary’ foods groups based on the 2013 Australian Dietary Guidelines [16,40]. The same categorisation method was used in previously published research [14]. The core foods group (*n* = 12 items) comprised of dairy, grains, fruits, vegetables, and meat and its alternatives; whilst the discretionary foods group (*n* = 20 items) comprised of foods with added fats and/or salt, and foods with added sugars. Additionally, the discretionary foods group was further categorised into sugary foods group (*n* = 18 items). The frequency (continuous data) of each item in the five individual core foods subgroups were summed to give the ‘total of the core food group intake’, and the frequency of each item in the two discretionary food groups were summed to give the ‘total of the discretionary food group intake’. This same method was used for sugar-containing items to give the ‘total of the sugary food group intake’. The focus of this study was on dietary trajectories of core, discretionary, and sugary foods, respectively. Children were included in the study if they had diet data available for at least three interview age points along with the clinical outcome data. 

#### 2.2.2. Other Predictors

Information on sociodemographic characteristics (including maternal age, marital status, country of birth, education level, employment status at 12 months postpartum, parity, and child gender), area-level socioeconomic status (SES), behavioural factors including maternal smoking practices during pregnancy, and biological factors including infant birth weight were collected via telephone interview at baseline (i.e., 8 weeks postpartum) using the adapted study questionnaire. Data on child age, breastfeeding duration, and age at which complementary (solid) foods were first introduced were periodically recorded via telephone interviews at 4 months, 8 months, 1 year, 2 years, and 3 years age points. Furthermore, children’s physical activity (time spent playing outdoors) was recorded at the 3-year interview [41]. 

#### 2.2.3. Anthropometric Data

Once the children reached the age of 3 years, they were invited (accompanied by their mothers) for a dental assessment. At the time of the dental visit, children’s weight and height were measured by trained and experienced health professionals using standardised methodology and equipment [42]. For weight measurement, a calibrated digital scale placed on a flat, hard surface was used, with children wearing light clothing and no shoes. The measured weight was recorded to the nearest 100 g. For the height measurement, a portable SECA stadiometer was used with a vertical backboard and movable headboard. The child’s head, back, buttocks, and heels were in contact with the vertical backboard. The measured height was recorded to the nearest 1 mm. For weight and height assessment, two measurements were taken by the examiner, and if the two measures for weight differed by more than 50 g, and/or if the two measures for height differed by more than 5 mm, then a third measurement was taken. For anthropometric outcome, age and gender-specific BMI z-score was calculated using the World Health Organization’s (WHO) (Geneva, Switzerland) AnthroPlus software program version 2.0 [43]. Children were categorised (based on the WHO age and gender specific cut-offs) as healthy (BMI z-score ≥ −2 and ≤+2 standard deviations (SD)), overweight (BMI z-score > +2 and ≤+3 SD) or obese (BMI z-score > +3 SD) [44]. For analytical purposes, overweight and obese categories were combined into a single category ‘overweight or obese’ (BMI z-score > +2 SD) due to the small number of obese children. 

#### 2.2.4. Dental Data

Children’s dental examinations were conducted between the ages of 3 and 4 years. The examinations were conducted by trained and experienced dental therapists in clinical settings using standardised protocols [45,46]. A standard dental index—decayed, missing, and filled tooth surfaces (dmfs)—was used to record the ECC prevalence [47]. For this study, ECC was characterised as the ‘presence of one or more decayed (non-cavitated or cavitated lesions), missing (due to decay), or filled tooth surfaces in any primary (baby) teeth in children less than 6 years of age’ [48]. 

### 2.3. Statistical Analyses

Statistical analyses were performed using Stata Statistical Software version 15.0 (StataCorp, College Station, TX, USA). Continuous data were presented as mean and SD and categorical data were presented as frequency and percentages. 

### 2.4. Dietary Pattern Analyses

#### 2.4.1. Group-Based Trajectory Modelling

Dietary patterns were examined using the Group-Based Trajectory Modelling (GBTM) analysis. A plug-in (PROC TRAJ) in Stata was used to construct the dietary trajectories. The GBTM uses finite mixture modelling and creates meaningful subgroups comprising of individuals who follow statistically similar trajectories. It statistically identifies (rather than assuming a priori) groups of distinctive trajectories that are summarised by a finite set of different polynomial functions of age or time, as determined by maximum likelihood estimation. The maximisation uses a general quasi-Newtown method. GBTM allows the trajectories to emerge from the data itself rather than establishing trajectories on the basis of an individual trait or traits. In terms of trajectory groups, this method determines the form and numbers that best fit the data. GBTM predicts the trajectory of each group, the form of each trajectory, estimates the probability for each individual for group membership, and assigns them to the group for which they have the highest probability. 

For GBTM, Bayesian information criteria (BIC) are often used for selecting the model (number of trajectory groups) that best represents the heterogeneity in the trajectories of the study sample. However, the BIC does not always clearly identify the ideal number of groups. Therefore, the objective of model selection is centred around summarising the data features in as parsimonious manner as possible. In this study analysis, a Poisson-based model was used due to the continuous distribution (count data) of the food frequency data at each age point. GBTM analysis comprises of a two-step process: (1) select the number of groups; and (2) determine the order of the polynomial defining each group’s trajectory (i.e., zero-order, linear, cubic, and quadratic). A series of 2- to 6-group models were fitted, starting with zero-order specifications for the trajectory shapes and moving to linear, cubic, and quadratic specifications until the best-fitting model (which was parsimonious and analytically tractable) was established. 

Prior to dietary analyses, the 32 food and drink items were categorised into meaningful groups (described earlier in the methods section) that had clinical relevance to the outcomes of obesity and ECC, respectively. Three food groups, namely core, discretionary, and sugary foods were used as input variables for GBTM. Trajectories of core foods were constructed due to their protective effect against obesity [19]. Trajectories of discretionary foods were constructed in relation to the obesity outcome, since foods high in saturated fats, salts, and sugars are known to be obesogenic [17]. Meanwhile, trajectories of sugary foods were generated in relation to the ECC outcome, since sugars are known to be one of the principal determinants of ECC [49]. Dietary data available for at least three interview periods were included in the analyses. In summary, dietary trajectories for ‘core’ and ‘discretionary’ foods were constructed for a total of 738 children, since they had the anthropometric data, whilst dietary trajectories for ‘sugary’ foods were constructed for a total of 718 participants, since they had ECC outcome data. 

#### 2.4.2. Predictors of Anthropometric Measures and ECC

Multi-level multivariable regression modelling was used for primary outcomes of overweight/obesity and ECC, respectively. Regression modelling for both outcomes was based on conceptual models; it was guided by prior evidence that certain dietary, biological, sociodemographic, socioeconomic, and behavioural factors were the likely candidate predictors [17,19,49,50]. Binary logistic regression was used to investigate the associations of dietary patterns (specifically ‘core’ and ‘discretionary’ foods) and other predictors with child weight status. Additionally, to investigate the associations of sugary foods dietary patterns and other predictors with the presence of dental caries; the countfit-function was used [51] to compare negative binomial and zero-inflated negative binomial regression. The negative binomial regression was the best fit, and hence, it was used for this analysis. 

For each primary outcome, a series of models was generated. The first model was generated with only the diet trajectory groups. Then, demographic factors were sequentially entered into the model to check for effect size and random variations. Furthermore, socioeconomic factors at the individual and area level were sequentially entered into the models followed by behavioural and biological factors, respectively. For this study, each research question is answered by presenting the full model with factors at all the levels. The fixed effects were presented as odds ratios (OR) and 95% confidence intervals (95 %CI) for child weight status, and incidence rate ratios (IRR) and 95 %CI for the prevalence of ECC. A significance level of 5% was used for all analyses.

### 2.5. Ethics Approval and Participant Consent

Ethics approval to conduct this study was given by the former Sydney South West Area Health Service—RPAH Zone (ID number X08-0115), Liverpool Hospital, University of Sydney and Western Sydney University. All participants signed a written consent form prior to study commencement. 

## 3. Results

Fifteen hundred mothers were invited to participate in the HSHK study, of whom 1035 formally agreed to participate (69% response rate). Participating (*n* = 1035) and non-participating mothers (*n* = 465) were compared on certain sociodemographic characteristics and chosen method of infant feeding. Both cohorts had no significant differences in terms of maternal age (Chi-square (*X*^2^) = 4.75, *p* = 0.153), educational level (*X*^2^ = 6.65, *p* = 0.328), and method of infant feeding (*X*^2^ = 2.46, *p* = 0.813). Before the baseline interview, a further 101 mothers either opted out or were non-contactable. Hence, in total, 934 mothers completed the interviews (62.2% response rate), and of these, 738 had the anthropometric outcome data (21% attrition rate), whilst 718 participants had the ECC outcome data (23% attrition rate). No differences in the age, education level, and method of infant feeding were evident between mothers who withdrew from the study and those who completed all the interviews, including the clinical assessments (data not reported).

‘Core’ and ‘discretionary’ foods intake trajectories were constructed for 738 children having anthropometric data (52% males and 48% females), while ‘sugary’ foods intake trajectories were constructed for 718 children having ECC data (52% males and 48% females). The mean (±SD) age of children was 3.57 (±0.25) years at the time of clinical assessment. Most children were of healthy weight (90%), with 7% being overweight, 3% having obesity, and 0.27% being underweight. Eleven percent (*n* = 38) of females and 9% (*n* = 34) of males were overweight or obese in the study sample. In relation to the prevalence of dental caries, 33% (*n* = 239) of children had ECC with a mean dmfs score of 1.96. There was no difference in the caries experience of male (*n* = 124) and female (*n* = 115) children. Furthermore, the majority of mothers were married and/or partnered, university educated, and non-smokers during pregnancy. The characteristics of participants in relation to the anthropometric and ECC outcomes are shown in Table 1 and Table 2, respectively.

### 3.1. Dietary Pattern Trajectories

#### 3.1.1. Core Foods

**Figure 1:**
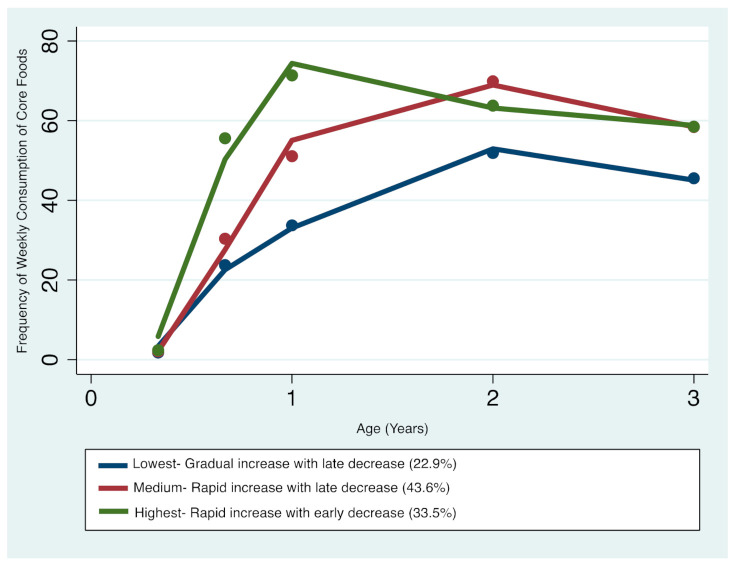
Trajectories of core foods consumption in infancy and early childhood.

Three distinct core foods trajectories were identified (Figure 1): trajectory 1 (Lowest consumers—gradual increase with late decrease) comprising 22.9% of the sample; trajectory 2 (Medium consumers—rapid increase with late decrease) comprising 43.6%; and trajectory 3 (Highest consumers—rapid increase with early decrease) comprising 33.5%. The resulting patterns indicate that children’s core foods intake increased between 4 months and 2 years of age, with frequency for all patterns decreasing between 2 and 3 years of age. From the age of 1 to 2 years, children with the highest consumption began to decrease their intake of core foods, while children in the lower consumption trajectories continued to increase their consumption until 2 to 3 years, after which a downward decline in core foods consumption was observed. Overall, the medium and highest trajectories seemed to converge with advancing age, whilst the lowest trajectory remained distinct at the 3-year age point (Figure 1).

#### 3.1.2. Discretionary Foods

**Figure 2:**
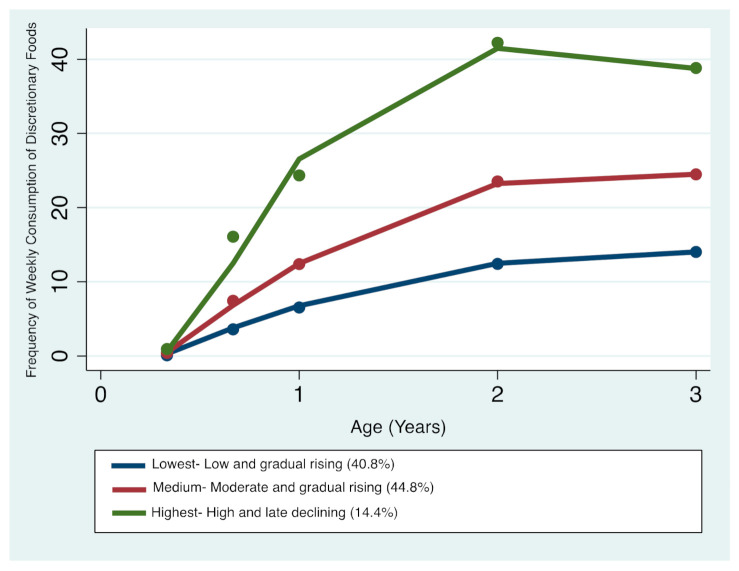
Trajectories of discretionary foods consumption in infancy and early childhood.

Three distinct discretionary foods trajectories were identified (Figure 2): trajectory 1—‘Lowest consumers—Low and gradual rising’ comprising 40.8% of the sample; trajectory 2—‘Medium consumers—Moderate and gradual rising’ comprising 44.8%; and trajectory 3—‘Highest consumers—High and late declining’ comprising 14.4%. The resulting patterns indicate that children’s discretionary foods intake steadily increased between 4 months and 3 years of age. Between 2 and 3 years of age, children who had the lowest and medium trajectories continued to have slightly higher or stable intakes respectively, whilst children who had the highest trajectories began a downward trend in discretionary foods consumption. Overall, all three trajectories remained distinctive with advancing age (Figure 2).

#### 3.1.3. Sugary Foods

**Figure 3:**
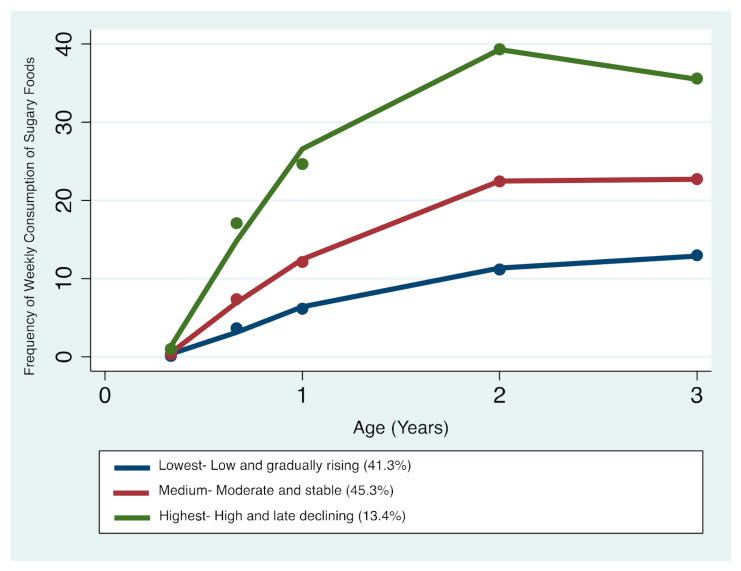
Trajectories of sugary foods consumption in infancy and early childhood.

Three distinct sugary foods trajectories were identified (Figure 3): trajectory 1—‘Lowest consumers—Low and gradual rising’ comprising 41.3% of the sample; trajectory 2—‘Medium consumers—Moderate and stable’ comprising 45.3%; and trajectory 3—‘Highest consumers—High and late declining’ comprising 13.4% of the total sample. The resulting patterns indicate that children’s sugary foods intake steadily increased between 4 months and 3 years of age. Between the ages of 2 and 3 years, children who had the lowest and medium trajectories continued to have slightly higher or stable intakes, respectively, whilst children who had the highest trajectories tended to begin a downward trend in sugar foods consumption. Overall, all the three trajectories remained distinctive with advancing age (Figure 3).

### 3.2. Impact of Dietary Pattern Trajectories and Other Predictors on Overweight/Obesity

After adjustment of covariates, the highest trajectory of discretionary foods intake compared with the lowest trajectory was independently associated with overweight or obesity (OR = 2.51, 95 %CI: 1.16–5.42; *p* = 0.019). Additionally, low area-level SES (deciles 1–2: most disadvantaged), compared with highest area-level SES (deciles 9–10: least disadvantaged) was associated with overweight or obesity (OR = 2.86, 95 %CI: 1.11–7.34; *p* = 0.029). There was no independent association between core food intake trajectories and overweight or obesity (Table 3).

### 3.3. Impact of Dietary Pattern Trajectories and Other Predictors on ECC

No statistically significant or clinically meaningful association was found between trajectories of sugary foods intake and ECC after adjusting for covariates (Table 4). In regard to other predictors, low area-level SES (deciles 3–4: highly disadvantaged—IRR = 2.02, 95 %CI: 1.08–3.77; *p* = 0.027 and deciles 1–2: most disadvantaged—IRR = 2.71, 95 %CI: 1.48–4.97; *p* = 0.001), compared with highest area-level SES (deciles 9–10: least disadvantaged) was associated with ECC. Furthermore, a longer duration of breastfeeding (≥52 weeks) was associated with ECC (IRR = 2.17, 95 %CI: 1.27–3.73; *p* = 0.005) compared with breastfeeding for 26 to 51 weeks (Table 4). 

## 4. Discussion

This study augments the existing literature on childhood nutrition by exploring the longitudinal trajectories of dietary intake in Australian children as they transition from infancy to early childhood using an innovative statistical approach such as GBTM and their association with two highly prevalent childhood health issues. 

In the present study, the prevalence of overweight and obesity was 10% while the prevalence of ECC was 33%. A similar birth cohort study based in South Australia reported 8.2% prevalence of overweight and obesity and 8.8% prevalence of ECC in children aged 24–36 months [28]. In an Australia-wide context, the prevalence of overweight and obesity in the present study is significantly lower (10% vs. 20%) [3], while the prevalence of ECC is identical (33% vs. 34%) [4]. 

This study investigated the association of longitudinal dietary trajectories with overweight or obesity and ECC at 3–4 years of age. A positive association between high frequency of discretionary foods intake (poor quality diet) and overweight or obesity was observed. However, no association was found between high frequency of sugary foods intake and ECC. Furthermore, no evidence of an inverse association between trajectories of core foods intake (good quality diet) and overweight or obesity was found. Nevertheless, the risk of childhood overweight or obesity and ECC was predicted by area-level SES, and the risk of ECC was predicted by child breastfeeding practices in expected directions.

The frequent consumption of energy-dense, nutrient-poor discretionary foods was prospectively associated with overweight and obesity. This finding is consistent with previous literature [52,53]. For instance, a previous systematic review identified a positive relationship between energy-dense, high-fat, and low-fibre dietary patterns, and later overweight and obesity risk based on high-quality prospective studies [54]. Another recent review including longitudinal cohort studies suggested that energy-dense diets increase the risk of obesity in childhood [55]. However, inconsistent findings have been reported, and a recent Australian cohort study did not find any association between unhealthy diet patterns and obesity in children aged 24–36 months [28]. Frequent consumption of discretionary foods can displace the intake of healthier core foods, provide excessive energy leading to weight gain, and ultimately contribute to incidence of chronic health conditions [56]. Hence, understanding the contribution of discretionary nutrients (such as saturated fat, salt, and added sugars) to total energy intake will assist in the identification of targets to focus our efforts on preschool children to improve their nutrient intakes, diet quality, and subsequent health.

Previous literature related to associations between unhealthy dietary patterns and anthropometric outcomes mostly includes older children and/or adolescents and/or is predominantly based on cross-sectional analysis using explanatory dietary pattern methods (e.g., factor analysis, PCA, and cluster analysis) [22,23,24]. To the best of our knowledge, this is the first Australian cohort study that uses the newly emerging GBTM approach to examine children’s diet patterns (or trajectories) in the first three years of life and investigates their impact on overweight/obesity between 3 and 4 years of age. 

For ECC, the present study did not find an association between sugary foods trajectories and ECC. This outcome is not surprising considering the inconsistent findings about the association between sugary diet patterns and dental caries measures reported in earlier studies [28,31]. A probable explanation for the lack of association in the present study might be that the dietary items, particularly sugary items, within the FFQ were limited, and the questionnaire assessed the dietary intake on a weekly basis. Moreover, the dietary assessment was self-reported. Nonetheless, a recent Australian cohort study also did not find any association between sugar-containing dietary patterns in the first 12 months and ECC at 24–36 months of age [28]. It was suggested that the lack of association was because 24–36 months of age might be too early to detect the impact of poor diet [28]. Likewise, another recent cohort study did not find any evidence for sugary diet patterns subsequently causing dental caries in Asian toddlers [31]. 

In relation to the methods of dietary assessment, FFQ is one of the most widely used instruments in epidemiological studies because of its relative simplicity, time efficiency, and cost-effectiveness [57]. However, a major disadvantage of FFQ is the reliance on self-reporting, which is influenced by participants’ honesty, education level, memory, and cognitive capability in terms of dietary intake. Such factors are suggested to significantly underestimate the true energy intake [58], thereby possibly affecting the true diet–disease relationship. On the contrary, objective data, for example SES (including education level, income, and employment status) is a composite indicator of health and is suggested to influence the dietary intake and diet quality, as well as the health of individuals. Thus, using such data helps to better explain the social inequalities in nutrition and health [59].

In the present study, beside dietary patterns, the two health outcomes were associated with certain socioeconomic and behavioural factors. Familial socioeconomic disadvantage was found to be a common risk factor for both overweight or obesity and ECC in the study sample. A recent systematic review identified familial wealth as one of the most important predictors for both obesity and ECC risk in preschool children [8]. The present study findings are consistent with previous literature that also found an inverse association between parental SES and children’s weight status and dental caries experience [60,61]. Poor parental income influences children’s dietary choices, since low-calorie, nutrient-rich foods (namely fruits, vegetables, and whole-grain cereals) are likely to be more expensive, therefore possibly leading to higher consumption of an energy-dense, nutrient-poor diet, which is relatively inexpensive [62]. Furthermore, a low family income may lead to limited access to a low-sugar diet, fluoridated toothpaste, and professional preventative measures [61], and subsequent poor dietary and lifestyle choices [62]. 

Breastfeeding is known to have considerable health benefits for both child and the mother [63]. However, this study found that a longer duration of breastfeeding (i.e., beyond 12 months of age) increases ECC risk. This finding adds to the current body of evidence about the causal role of breastfeeding on ECC. Previous systematic reviews [64,65] reported that the risk of ECC increases with breastfeeding beyond 12 months of age. Although a recent Australian cohort study [66] did not find an association between breastfeeding beyond 12 months of age and ECC, the authors concluded that the size and direction of the relationship was suggestive of a higher risk. Meanwhile, a second national Australian study found that this effect was modified by fluoride, and an association between ECC and breastfeeding beyond 24 months was only evident in children without access to a fluoridated water supply [67]. Breastfeeding is known to be protective against caries in the first 6 to 12 months of life compared to no breastfeeding, but ECC prevalence tends to increase if breastfeeding continues beyond the first year of life [64,65]. The exact age at which breastfeeding begins to have a cariogenic effect is not known, which is possibly because studies have used different cut-offs, e.g., 12 months [68], 18 months [69], and 24 months and beyond [70]. Human breast milk is suggested to be more cariogenic than bovine milk but less than infant formula [71]. In the first 12 months, infants are usually fed either breastmilk or formula, both of which have almost the same carbohydrate content [65]. After 12 months, infants are usually weaned onto cow’s milk, which has a significantly lower carbohydrate content than both formula and human milk. Hence, a prolonged contact of teeth with human milk combined with intake of sugars in the diet leads to an acidogenic oral environment and subsequent demineralisation of tooth (or teeth) due to the bacterial fermentation of sugars [72]. Furthermore, these elements can be modified by various risk factors such as SES, maternal education, maternal smoking, parity, sugar intake, fluoride exposure, and oral hygiene practices [73]. Some studies have also shown that children who are breastfed for longer durations tend to consume cariogenic foods more frequently [68,74]. 

### Strengths and Limitations

This study used the longitudinal data from the HSHK birth cohort study, thereby overcoming the possibility of reverse directionality between dietary patterns and overweight/obesity and ECC outcomes [28]. Furthermore, oral health related birth-cohort studies such as HSHK are rare, and this study provides an opportunity to explore the common risk factors of two highly prevalent health issues affecting children at present. This study examines the frequency of intake of core foods, discretionary, and added-sugar foods over a longitudinal period in early years of life and its impact on obesity and ECC. Earlier studies have primarily focussed on assessing dietary patterns in older children or adults [24,25]. The dietary intake was able to be estimated at multiple and regularly spaced time points over the first three years of life, thus allowing for the influence of dietary patterns to possibly manifest into examinable changes in weight status and teeth at 3 to 4 years of age. The dietary patterns were generated using the newly emerging Group-Based Trajectory Modelling (GBTM) in longitudinal and clinical research, which provides a comprehensive picture of the evolution of children’s diet over time. Additionally, the use of multiple meaningful dietary patterns (i.e., core, discretionary, and sugar-based) to assess relationships with obesity and ECC outcomes is unique and a major strength of the present study. The attrition rate of the study sample by the time of clinical assessment (i.e., at 3–4 years of age) was considerably lower than those of other birth cohort studies [28,75]. Various sociodemographic, socioeconomic, behavioural, and biological predictors were also assessed, which would assist in identifying potential target groups for preventing two health outcomes using the common risk factor approach in health promotion. 

This study also has some limitations that need to be acknowledged. First, the dietary intake was based on parent reports through interviews and food frequency questionnaire (FFQ; therefore, there is a possibility of underreporting, inaccurate dietary recall, and/or social desirability bias. However, FFQ are commonly used in longitudinal studies because they are cost-effective and relatively quick to complete [57]. This assisted in maintaining good retention in the present study. In addition, since the data were longitudinal, the chances of heaping of data and recall bias are minimised. Second, a FFQ adapted from well-established literature was used in the study; however, it was not able to capture the whole diet. Moreover, the short FFQ consisted of essential core and discretionary foods listed in the Australian dietary guidelines; however, the items’ list is limited, particularly in relation to foods with free sugars (a strong determinant of ECC) [66], which might have produced different dietary trajectories. Similarly, in regard to core foods, refined cereals could not be distinguished from unrefined cereals; therefore, popular cereals that are high in sugar were categorised as core foods rather than discretionary foods [76]. Additionally, only the frequency of intake of foods was recorded rather than their frequency, amount, and relative percent of total calories, so we could not capture actual dietary intake, since the amount might vary within and between individuals, and total calories might vary between different foods. Another potential limitation is that the influence of parental BMI on children’s weight status could not be evaluated, considering that this is one of the most important determinants of childhood overweight and obesity. Furthermore, in relation to the predictors of ECC, the frequency and timing of breastfeeding (day- and night-time), and role of oral hygiene practices such as supervised tooth brushing, frequency of toothbrushing, and use of fluoridated toothpaste could not be evaluated, which might have produced different results. 

## 5. Conclusions

In summary, this is one of the first studies to describe the early life dietary patterns using Group-Based Trajectory Modelling (GBTM) and examine their impact on two highly prevalent chronic diseases in childhood—obesity and early childhood caries (ECC). An independent association was found between the highest trajectory of discretionary foods intake and being overweight or obese. However, an association between trajectories of sugary foods intake and ECC could not be established. Further research to investigate the impact of longitudinal trajectories of free-sugars intake on ECC is warranted. Familial socioeconomic disadvantage was identified to be a common risk factor for both health conditions, thus justifying the concept of common risk factor approach (CRFA) in disease epidemiology and prevention. Additionally, breastfeeding beyond 12 months was found to be a significant predictor of ECC in this sample; however, other factors related to breastfeeding (frequency and timing) and oral hygiene practices were unadjusted. In conclusion, these study findings have identified target groups to implement preventative and interventional strategies against the rising obesity and dental caries burden in early childhood.

## Figures and Tables

**Table 1:** Participant characteristics—diet trajectories, demographic, socioeconomic, behavioural, and biological characteristics based on anthropometric groups (*n* = 738).

Characteristics	Healthy ^a^ (*n* = 666)	Overweight and Obese ^b^ (*n* = 72)
Diet trajectories		
Core foods		
Lowest (Gradual increase with late decrease)	149 (89.76%)	17 (10.24%)
Medium (Rapid increase with late decrease)	288 (88.62%)	37 (11.38%)
Highest (Rapid increase with early decrease)	229 (92.71%)	18 (7.29%)
Discretionary foods		
Lowest (Low and gradual rising)	282 (93.69%)	19 (6.31%)
Medium (Moderate and stable)	297 (89.46%)	35 (10.54%)
Highest (High and late declining)	87 (82.86%)	18 (17.14%)
Demographic factors		
Child age (in years) Mean ±SD	3.57 ± 0.25	3.58 ± 0.27
Child gender		
Male	350 (91.15%)	34 (8.85%)
Female	316 (89.27%)	38 (10.73%)
Maternal age (in years) Mean ± SD	31.54 ± 5.02	30.64 ± 6.58
Maternal marital status		
Married	616 (90.32%)	66 (9.68%)
Single	50 (89.89%)	6 (10.71%)
Maternal country of birth		
Australia-born	309 (9.09%)	34 (9.91%)
English speaking country	36 (90%)	4 (10%)
Non-English-speaking country	321 (90.42%)	34 (9.58%)
Number of children in household		
1	334 (90.27%)	36 (9.73%)
2	210 (90.52%)	22 (9.48%)
≥3	122 (89.71%)	14 (10.29%)
Individual-level socioeconomic status		
Maternal education		
University	314 (92.90%)	24 (7.10%)
College/TAFE	119 (89.47%)	14 (10.53%)
Completed 12	132 (88.59%)	17 (11.41%)
Left school < 12	101 (85.59%)	17 (14.41%)
Maternal work status		
Not working	339 (88.74%)	43 (11.26%)
Working	298 (91.98%)	26 (8.02%)
Area-level socioeconomic status		
Index of relative socioeconomic advantage and disadvantage		
Deciles 9–10	162 (95.29%)	8 (4.71%)
Deciles 7–8	123 (92.48%)	10 (7.52%)
Deciles 5–6	18 (90%)	2 (10%)
Deciles 3–4	157 (88.70%)	20 (11.30%)
Deciles 1–2	206 (86.55%)	32 (13.45%)
Behavioural factors		
Breastfeeding duration		
<17 weeks	236 (87.41%)	34 (12.59%)
17–25 weeks	71 (89.87%)	8 (10.13%)
26–51 weeks	165 (90.16%)	18 (9.84%)
≥52 weeks	193 (94.15%)	12 (5.85%)
Age of introduction of solid foods		
<17 weeks	63 (85.14%)	11 (14.86%)
17–25 weeks	362 (90.05%)	40 (9.95%)
≥26 weeks	232 (91.70%)	21 (8.30%)
Infant feeding at 4-weeks age		
Only BF	436 (92.77%)	34 (7.23%)
Only FF	87 (83.65%)	17 (16.35%)
Both BF and FF	143 (87.20%)	21 (12.80%)
Outdoor physical activity		
≥180 min	414 (89.61%)	48 (10.39%)
<180 min	239 (91.22%)	23 (8.78%)
Maternal smoking during pregnancy		
No	636 (90.60%)	66 (9.40%)
Yes	30 (83.33%)	6 (16.67%)
Biological factors		
Infant birthweight		
Normal/High	634 (90.44%)	67 (9.56%)
Low	32 (86.49%)	5 (13.51%)

^a,b^ The total of the categories might not always add up to 738 due to missing or incomplete data for some items. Index of relative socioeconomic advantage and disadvantage: deciles 9–10 = least disadvantaged; deciles 7–8 = low disadvantaged; deciles 5–6 = moderately disadvantaged; deciles 3–4 = highly disadvantaged; deciles 1–2 = most disadvantaged. SD: standard deviation. *n*: sample size. BF: breastfeeding. FF: formula feeding.

**Table 2:** Participant characteristics—diet trajectories, demographic, socioeconomic, and behavioural characteristics based on early childhood caries (ECC) groups (*n* = 718).

Characteristics	ECC − ve ^a^ (*n* = 479)	ECC + ve ^b^ (*n* = 239)
Diet trajectories		
Sugary foods		
Lowest (Low and gradual rising)	219 (67.80%)	104 (32.20%)
Medium (Moderate and stable)	194 (65.54%)	102 (34.46%)
Highest (High and late declining)	66 (66.67%)	33 (33.33%)
Demographic factors		
Child age (in years) Mean ± SD	3.56 ± 0.25	3.59 ± 0.25
Child gender		
Male	248 (66.67%)	124 (33.33%)
Female	231 (66.76%)	115 (33.24%)
Maternal age (in years) Mean ±SD	31.54 ± 5.09	31 ± 5.49
Maternal marital status		
Married	442 (66.77%)	220 (33.23%)
Single	37 (66.07%)	19 (33.93%)
Number of children in household		
1	257 (71.59%)	102 (28.41%)
2	146 (64.60%)	80 (35.40%)
≥3	76 (57.14%)	57 (42.86%)
Individual-level socioeconomic status		
Maternal education		
University	223 (69.25%)	99 (30.57%)
College/TAFE	97 (75.78%)	31 (24.22%)
Completed 12	94 (62.67%)	56 (37.33%)
Left school < 12	65 (55.08%)	53 (44.92%)
Maternal work status		
Not working	233 (62.47%)	140 (37.53%)
Working	226 (72.20%)	87 (27.80%)
Area-level socioeconomic status		
Index of relative socioeconomic advantage and disadvantage		
Deciles 9–10	122 (78.21%)	34 (21.79%)
Deciles 7–8	88 (69.84%)	38 (30.16%)
Deciles 5–6	16 (76.19%)	5 (23.81%)
Deciles 3–4	116 (65.91%)	60 (34.09%)
Deciles 1–2	137 (57.32%)	102 (42.68%)
Behavioural factors		
Breastfeeding duration		
<17 weeks	176 (66.17%)	90 (33.83%)
17–25 weeks	54 (71.05%)	22 (28.95%)
26–51 weeks	129 (72.88%)	48 (27.12%)
≥52 weeks	119 (60.10%)	79 (39.90%)
Maternal smoking during pregnancy		
No	453 (66.42%)	229 (33.58%)
Yes	26 (72.22%)	10 (27.78%)

^a,b^ The total of the categories might not always add up to 718 due to missing or incomplete data for some items. Index of relative socioeconomic advantage and disadvantage: deciles 9–10 = least disadvantaged; deciles 7–8 = low disadvantaged; deciles 5–6 = moderately disadvantaged; deciles 3–4 = highly disadvantaged; deciles 1–2 = most disadvantaged. SD: standard deviation. *n*: sample size. ECC: early childhood caries.

**Table 3:** Final model—impact of diet trajectories, demographic, socioeconomic, behavioural, and biological factors on overweight or obesity in early childhood.

	Adjusted OR	95% CI		*p*-Value	Overall *p*-Value
Diet trajectories					
Core foods					
Lowest (Gradual increase with late decrease)	ref				0.559
Medium (Rapid increase with late decrease)	1.26	0.64	2.47	0.505	
Highest (Rapid increase with early decrease)	0.82	0.39	1.72	0.603	
Discretionary foods					
Lowest (Low and gradual rising)	ref				0.022
Medium (Moderate and stable)	1.55	0.83	2.89	0.165	
Highest (High and late declining)	2.51	1.16	5.42	0.019	
Demographic factors					
Child age (in years)	1.03	0.37	2.86	0.958	0.973
Child gender					
Male	ref				0.496
Female	1.21	0.72	2.06	0.468	
Maternal age (in years)	1.00	0.95	1.06	0.856	0.803
Maternal marital status					
Married	ref				0.521
Single	0.68	0.24	1.89	0.457	
Maternal country of birth					
Australia-born	ref				0.252
English speaking country	1.23	0.38	3.99	0.734	
Non-English-speaking country	0.71	0.38	1.31	0.275	
Number of children in household					
1	ref				0.346
2	0.80	0.43	1.48	0.481	
≥3	0.67	0.31	1.45	0.314	
Individual-level socioeconomic status					
Maternal education					
University	ref				0.707
College/TAFE	1.11	0.52	2.39	0.777	
Completed 12	1.06	0.51	2.24	0.861	
Left school < 12	1.19	0.51	2.80	0.678	
Maternal work status					
Not working	ref				0.381
Working	0.77	0.43	1.37	0.375	
Area-level socioeconomic status					
Index of relative socioeconomic advantage and disadvantage					
Deciles 9–10	ref				0.030
Deciles 7–8	1.65	0.60	4.49	0.328	
Deciles 5–6	1.94	0.35	10.83	0.449	
Deciles 3–4	2.25	0.87	5.81	0.092	
Deciles 1–2	2.86	1.11	7.34	0.029	
Behavioural factors					
Breastfeeding duration					
26–51 weeks	ref				0.121
<17 weeks	0.63	0.29	1.38	0.257	
17–25 weeks	0.65	0.23	1.89	0.433	
≥52 weeks	0.55	0.25	1.22	0.143	
Age of introduction of solid foods					
<17 weeks	ref				0.492
17–25 weeks	0.97	0.41	2.28	0.948	
≥26 weeks	0.88	0.34	2.25	0.786	
Infant feeding at 4-weeks age					
Only BF	ref				0.072
Only FF	1.96	0.81	4.75	0.137	
Both BF and FF	1.85	0.96	3.56	0.067	
Outdoor physical activity					
≥180 mins	ref				0.730
<180 min	0.89	0.51	1.58	0.714	
Maternal smoking during pregnancy					
No	ref				0.822
Yes	1.07	0.31	3.69	0.908	
Biological factors					
Infant birthweight					
Normal/High	ref				0.730
Low	1.31	0.45	3.78	0.621	

Index of relative socioeconomic advantage and disadvantage: deciles 9–10 = least disadvantaged; deciles 7–8 = low disadvantaged; deciles 5–6 = moderately disadvantaged; deciles 3–4 = highly disadvantaged; deciles 1–2 = most disadvantaged. AOR: adjusted odds ratio. 95% CI: 95% confidence interval. ref: reference category. BF: breastfeeding. FF: formula feeding.

**Table 4:** Final model—impact of diet trajectories, demographic, socioeconomic, and behavioural factors on early child- hood caries.

	Adjusted IRR	95% CI		*p*-Value	Overall *p*-Value
Diet trajectories					
Sugary foods					
Lowest (Low and gradual rising)	ref				0.737
Medium (Moderate and stable)	1.30	0.85	2.00	0.228	
Highest (High and late declining)	0.90	0.47	1.70	0.747	
Demographic factors					
Child age (in years)	0.87	0.39	1.97	0.747	0.941
Child gender					
Male	ref				0.246
Female	0.86	0.57	1.29	0.473	
Maternal age (in years)	0.98	0.94	1.03	0.464	0.755
Maternal marital status					
Married	ref				0.352
Single	1.34	0.59	3.00	0.482	
Number of children in household					
1	ref				0.563
2	1.11	0.69	1.78	0.654	
≥3	1.53	0.84	2.79	0.164	
Individual-level socioeconomic status					
Maternal education					
University	ref				0.195
College/TAFE	0.75	0.43	1.33	0.329	
Completed 12	1.07	0.60	1.89	0.817	
Left school < 12	1.75	0.91	3.37	0.092	
Maternal work status					
Not working	ref				0.126
Working	0.78	0.52	1.18	0.241	
Area-level socioeconomic status					
Index of relative socioeconomic advantage and disadvantage					
Deciles 9–10	ref				0.005
Deciles 7–8	1.64	0.86	3.13	0.132	
Deciles 5–6	0.67	0.19	2.28	0.521	
Deciles 3–4	2.02	1.08	3.77	0.027	
Deciles 1–2	2.71	1.48	4.97	0.001	
Behavioural factors					
Breastfeeding duration					
26–51 weeks	ref				0.008
<17 weeks	1.23	0.72	2.08	0.448	
17–25 weeks	0.99	0.47	2.06	0.976	
≥52 weeks	2.17	1.27	3.73	0.005	
Maternal smoking during pregnancy					
No	ref				0.223
Yes	0.51	0.17	1.56	0.242	

Index of relative socioeconomic advantage and disadvantage: deciles 9–10 = least disadvantaged; deciles 7–8 = low disadvantaged; deciles 5–6 = moderately disadvantaged; deciles 3–4 = highly disadvantaged; deciles 1–2 = most disadvantaged. Adjusted IRR: adjusted incidence rate ratio. 95% CI: 95% confidence interval. ref: reference category.

## Data Availability

The data of this study cannot be shared publicly due to the presence of sensitive (confidential) participants’ information.

## Supplementary Materials

The following are available online at https://www.mdpi.com/article/10.3390/nu13072240/s1, Table S1: List of dietary items (*n* = 32) recorded in the short food frequency questionnaire.
