# Supplementary material for: Impact of Dietary Trajectories on Obesity and Dental Caries in Preschool Children: Findings from the Healthy Smiles Healthy Kids Study
Source: Nutrients. 2021 Jun 29;13(7):2240. doi: 10.3390/nu13072240 (PMC8308427; doi:10.3390/nu13072240)
Supplement: Supplementary file 1 [file nutrients-13-02240-s001.zip › nutrients-1240545-supplementary.pdf]

Supplementary Material

Table S1. List of dietary items (n = 32) recorded in the short food frequency questionnaire.

**Dietary Items**	**Interview Phases**				
(In the past 7 days how often (count data) was your baby/child fed each of the following foods and/or drinks?)	**4-months**	**8-months**	**1-year**	**2-years**	**3-years**
**Core (n = 12)**					
Dairy					
Cow milk					
Other milk: soy milk, goat milk, rice milk					
Plain Yoghurt					
Cheese					
Grains					
Baby cereal					
Cereals					
Other starches (e.g., breakfast cereals, bread, rice, pasta, crackers)					
Fruits					
Vegetables					
Meat and alternatives					
Meat, chicken, combination dinners					
Fish or shellfish					
Eggs					
**Discretionary (n = 20)**					
Foods with added sugars					
Flavored milk (e.g., Milo™, Nesquik™, Chocolate milk)					
Flavored yogurt					
Ice cream, custard and other dairy desserts					
Fruit Juice (if diluted, state the %)					
Soft drinks (e.g., Coke™)					
Cordial including Ribena™					
Sports drinks (e.g., PowerAde™ and Gotarade™)					
Powdered drink (e.g., Tang™)					
Flavored mineral water					
Iced Tea					
Iced Coffee					
Hot Tea (if sugar added)	X	X	X		
Hot Coffee (if sugar added)	X	X	X		
Lollies (candy), chocolate and sugar-based confectionary					
Syrups, jams and sweet spreads (e.g., Nutella, honey and maple syrup)					
Honey				X	X
Packaged sweet snacks e.g., muesli bars/fruit straps	X	X	X		
Biscuits, cakes and/or puddings					
Foods with added fats and/or salt					
French fries/ hot chips					
Packed snacks such as Potato chips/crisps	X	X	X		

X: Frequency of intake NOT recorded at that specific interview phase.
